# Cognitive mapping: Enhancing discussions in the transdisciplinary design and health research space?

**DOI:** 10.1080/14606925.2025.2510800

**Published:** 2025-06-26

**Authors:** Alastair S. Macdonald

**Affiliations:** The Glasgow School of Art, Glasgow, Scotland

**Keywords:** Cognitive maps, transdisciplinary research, design and health research space

## Abstract

This paper explores the potential for cognitive maps to enhance discussion of transdisciplinary research (TDR). Differentiating between tacit, internalised mental maps and sharable, visual cognitive maps as his point of departure the author revisits Sanders' seminal 2006 cognitive map modelling the topography of design research and practice. He then articulates a rationale for creating a new framework for mapping the collaborative design and health research space, discussing how this may allow for the exploration of common research goals but approached from alternative but potentially mutually complementary perspectives, drawing from—and perhaps combining—the preferred methods and epistemes of different fields. A set of mappings of four different approaches to a particular health challenge is then discussed in relation to three identified characteristics of TDR, revealing options for design's contribution working in collaboration with other disciplines. (136)

## Introduction

There has been increasing encouragement for transdisciplinary research (TDR), occurring at the interface between different disciplines, to address complex global and societal challenges, such as antimicrobial resistance (UKRI 2023) or to achieve net zero (UKRI 2024). TDR has been described as 'a context-driven and problem-focused approach to knowledge production that involves collaboration across scientific disciplines and academic and non-academic sectors' (Yeung et al. 2021, 1–2).

### Why cognitive maps?

A common issue as teams coalesce around a TDR opportunity is that each member may bring with them tacitly their own field's preferred methods and approaches and a relative ignorance of others'. Cognitive maps may assist in revealing these individual predilections. Tversky (1993) uses the analogy of topographic maps when discussing cognitive maps, as visually representing relationships between social knowledge, similar to how features are located and shown spatially within a map. Shen et al. distinguish between mental models, 'individuals' internal representation of perceived reality, which help individuals to make sense of and give meaning to the world around them' (Shen, Tan, and Siau 2019, 281) and cognitive maps 'externalized portrayals of mental models in visuospatial layout' (Shen, Tan, and Siau 2019, 281). This distinction between mental maps, i.e. internal, tacit knowledge, and cognitive maps, i.e. enabling knowledge to be made explicit and shareable, has salience for TDR, particularly in their potential for identifying, developing an understanding of, and suggesting options for approaches and methods from fields perhaps less familiar to one's own.

As his point of departure, the author revisits Sanders (2006) seminal cognitive map used to model the topography of design research and practice. Inspired by her example but with a different intention and, drawing from his experience of TDR involving the design and health research communities, the author ventures that cognitive maps may enhance the exploration of common goals in TDR through helping to consider and discuss alternative approaches and methods. By way of example, he firstly illustrates one rationale for the construction of a framework for the mapping of the collaborative design and health research space, and then retrospectively examines a set of four examples to highlight the potential for alternative approaches to addressing a particular health challenge. These mappings suggest that design, collaborating with other disciplines, and introducing alternative approaches and methods may offer—in certain instances—distinctive but less familiar contributions, perhaps not fully appreciated by fields normally associated with a specific health challenge. Finally, discussing these examples against Yeung et al. (2021) three identified characteristics for TDR, he proposes that the use of such mappings may be useful at the early conceptualisation stages and planning stages to achieve the characteristics of TDR.

## Sanders' map

With her background in anthropology, experimental and quantitative psychology and her extensive experience of a wide range of design methods and approaches, Sanders (2006) proposed a cognitive map for the design community. Modelling the framework for the design research space as a 'scaffolding for thinking and talking about design research', Sanders' intent was to make sense of the 'jumble of approaches' and for visualising relationships between methods. In this, and in subsequent work with Stappers (e.g. Sanders and Stappers 2008), Sanders' idea was 'to view the design research space as a landscape and to give it a visual representation borrowing from the elements of the maps that we may have in our minds … to find our way around places' (Sanders 2006, 4). In her 2006 article, Sanders distinguished between 'cognitive collages' and 'cognitive maps', referencing a discussion in Tversky (1993), later reverting to the term 'maps'. For the author's purposes 'maps' and 'mappings' are the preferred terms used throughout.

**Figure 1.**
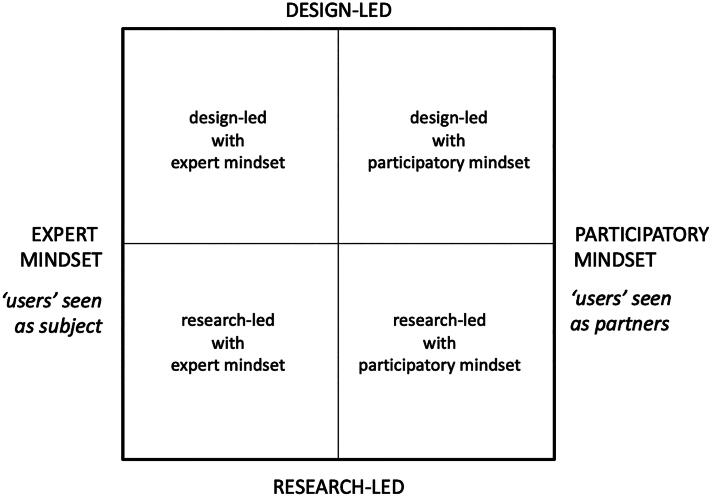
This figure shows the two axes and four quadrants of Sanders (2006, 2008). The horizontal axis has expert mindset to the left and participatory mindset to the right. The vertical axis has research-led at the base and design-led at the top. Clockwise from the top-left quadrant are design-led with expert mindset, design-led with participatory mindset (top right), research-led with participatory mind-set (bottom right), and research-led with expert mindset (bottom left). Reproduced by kind permission of Liz Sanders.

Sanders situated design's different approaches and research fields in relation to each other through a matrix derived from two axes distinguishing types of mindset from types of approach. Mindset forms the horizontal 'X' axis with, to the left, expert-mindset (users seen as subjects—reactive informers) and, to the right, participatory-mindset (users seen as partners—active co-creators). Forming the vertical 'Y' axis is approach, with research-led at the base and design-led at the top. This matrix arrangement resulted in the four quadrants as detailed in Figure 1.

**Figure 2.**
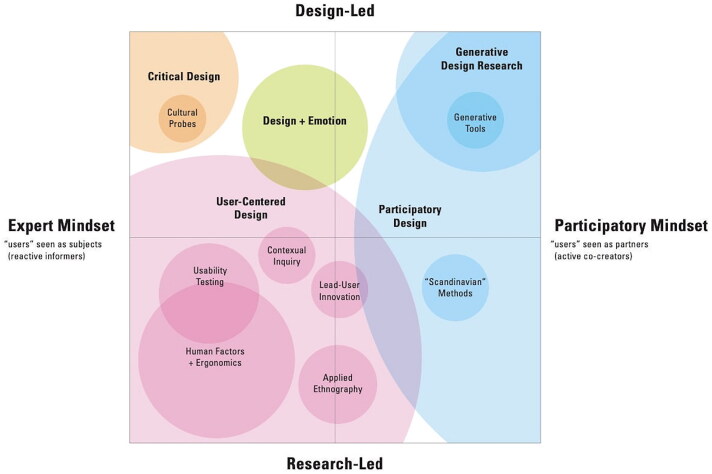
Sanders' 'evolving map of design practice and design research' (Sanders 2008, 3). Reproduced by kind permission of Liz Sanders.

Sanders, in further diagrams (e.g. Figure 2), populated this map locating, e.g. critical design and cultural probes in the top left corner (design-led with expert mindset), and generative design research in the top right quadrant (design-led with participatory mindset). Human factors sit within the more expansive territory of user-centred design in the bottom left quadrant (research-led with expert mindset), and participatory design occupies most of the top right (design-led with participatory mindset) as well as the bottom right (research-led with participatory mindset) quadrants.

In further articles, e.g. Sanders (2008), Sanders and Stappers (2008), the points of emergence and the relative pre-occupations of various types of research methods and disciplines were detailed, drawing from Sanders' extensive experience of and familiarity with the depicted fields. In doing so, she referenced the evolution of traditional and emerging design practices, distinguishing the changing roles of the user, the researcher and the professional designer, the (then) new domain of collective creativity with reference to developments in co-creation, co-design and participatory design and the coming together of the more user-centred (US) and more participative (Scandinavian) approaches. Sanders acknowledged that 'the underlying landscape of the map may be relatively permanent, changing only as major forces affect it' (Sanders 2008, 13). As new tools and methods emerged, and working with Stappers, Sanders invited the design research community to engage in a conversation about this design research space and to add to this collective cognitive map. Since Sanders first published her map, it has proved a seminal point of reference for designers, design researchers and doctoral students discussing the design research territory and the different approaches to, or types of design research methods, tools and specialisms within this.

### The evolving design research space

Sanders' original map is approaching two decades old. Meanwhile, the field of design has continued to evolve. For example, the design and health fields have collaborated to a significant extent with critical reflections on the mutual value of each other's approaches and methods. These include discussion of, e.g. the value of design thinking and methods to health studies (e.g. Oliveira, Zancul, and Fleury 2021), the challenges and tensions facing designers involved in health research (Groeneveld et al. 2018; Craig, Reay, and Nakarada-Kordic 2019), how design thinking and methods support interdisciplinary solutions (Andrawes, Johnson, and Coleman 2021), or the strengths and weaknesses of forms of co-design in health with—or without—the involvement of designers (Donetto, Tsianakas, and Robert 2014; Robert and Macdonald 2017). There has also been a major survey of the extent of design theory and practice evident within the context of health (Chamberlain et al. 2015), the establishment of a specific journal for the field (Taylor and Francis 2024), and the establishment of an international conference series (Lab4Living 2004).

## Common concerns, tacit differences?

Given this growing collaboration between the design and health fields, to what extent are the theoretical and epistemological bases, tacit knowledge, expertise and preferred practices, approaches and methods of one field understood by the other? Some established teams will have built up close working relationships, and have a tacit, if not an explicit, understanding of how their approaches and methods will mesh in the design of a programme of TDR. Others, however, will be unfamiliar with or new to this type of collaboration. With the increasing push for TDR, and with further fields adding to those from design and health, would a way of explicitly mapping—externalising—each field's approaches and methods be useful in the early conceptual stages of TDR? Inspired by Sanders' example but with the intention of illustrating how a mapping framework for the TDR design and health space might be constructed, the author now discusses how two preoccupations key to both fields—modes of engagement and types of evidence—suggest one option for a mapping of that space.

### Modes of engagement

With the contemporary policy turn towards co-production and co-research, the use of various forms of and approaches to collaborative practice has increased across many areas of design and health research and practice. Collaborative design (co-design) has its roots in the 1970s Scandinavian Participatory Design movement whose approaches have been widely discussed, adopted and adapted in academic and practice-based design communities, reflecting the increasing democratization of the activity of designing. The various 'co' terms have been the subject of much discussion, e.g. the co-creation of services (Cottam and Leadbetter 2004), the use of experience-based co-design (EBCD) in healthcare quality improvement (Donetto et al. 2015; Robert et al. 2021), and the co-production of healthcare services (Batalden et al. 2016). The term co-design suggests that designing is an innate, latent skill facilitated through appropriate conditions, spaces, types of practice and materials. Indeed, one might accept the view proposed by Choukeir (2021) that 'we are all designers …all that we do, almost all the time, is design, for design is basic to all human activity', a facility Gorb and Dumas refer to as silent design (1987, 152). This is the basic premise of EBCD which presents a potentially interesting challenge for designers, particularly given it is a very successful and proven co-design-without-designers approach, which has made considerable progress within healthcare. '[EBCD] is an approach that enables staff and patients (or other service users) to co-design services and/or care pathways, together in partnership' (Point of Care Foundation 2018). Donetto, Tsianakas, and Robert (2014) summarized a decade of EBCD's achievements in improving patient experiences, updated with a synthesis of the EBCD literature by Francis-Auton et al. (2024). This form of designing has been able to achieve what has proved and continues to prove a challenge for design in terms of the consistent application, repeatability, refinement and scaling of a method. This potentially problematises the role of designers, and poses interesting questions and challenges for design, a discussion developed in Macdonald (2017). However, if co-design-without-designers approaches can exist within healthcare, can one differentiate what might constitute 'designerly' effects (i.e. those arising from the approaches and methods of a professionally trained designer) as distinct from 'design-like' effects (i.e. design methods and approaches used by non-designers, in this case healthcare professionals and patients) in co-design approaches? The above tends to suggest there is some common ground in design and health approaches, through co-design, albeit with their respective differences. Robert and Macdonald (2017) develop this discussion, highlighting the strengths, weaknesses, and different types of outcomes that each approach may tend to result in. For example, EBCD may result in a more limited range of outcomes, more incremental and less radical solutions but have measurable economic benefits, well-reported evidence and solutions that are scalable, whereas design is iterative, makes ideas tangible early, tends to create bespoke experience prototypes that are problematic to scale and adapt, often without economic evaluation and with poorly reported evidence.

Here, it is also important to differentiate approaches that are merely consultative from those that are fully participative, involving different degrees of 'people power' (Horne, Khan, and Corrigan 2013). Savory (2010) distinguishes between, e.g. research '*on'* and research led '*by'* the patient and the public through four strategies for incorporating patient and public involvement (PPI) in translative healthcare research, the strategy selected dependent on the purpose of the participation. Other frameworks have also made clear similar shifts in doing '*to'*, '*for'* and '*with'*, such as Arnstein's Ladder of Citizen Participation (1969); this commences with 'manipulation' and progresses through stages such as 'consultation' and 'partnership', eventually to 'citizen control'. In a more contemporary interpretation of Arnstein's model, the New Economics Foundation's 'Ladder of Co-production' (2014, 85) delineates the progressive and increasingly commonly adopted shift from '*doing to*' (coercion, education), through '*doing for'* (informing, consultation and engagement), to '*doing with*' (co-design, co-production). While an intervention for a randomised controlled trial (RCT) may be developed collaboratively *with* individuals (such as with survivors of a condition or those with lived experience), the RCT is normally required to be administered *to* individuals.

### Evidence?

Evidence is a *sine qua non* for service quality improvement in health. However, what constitutes evidence is subject to debate. Petticrew and Roberts discuss a 'hierarchy of evidence' framework for public health based on a typology of evidence matrix with eight key features, which 'emphasises the need to match research questions to specific types of research' (2003). However, Glasby, Walshe and Harvey take the view that prevailing approaches to evidence-based practices are 'too dominated by formal (often medical) research and by traditional research hierarchies, which prioritise quantitative research (particularly systematic reviews and randomised controlled trials) over other ways of knowing the world' (2007a, 325). They argue that there are a number of very different ways to generate valid knowledge, that none offers a definite insight into a particular issue, and that there is a need for evidence of different types from multiple sources to be synthesised and integrated. Indeed, Lewin, Glenton and Oxman argue, in the case of complex healthcare interventions, that these 'involve social processes that can be difficult to explore using quantitative methods alone' (2009). Glasby, Walshe and Harvey propose a useful, non-hierarchical typology of evidence arguing that, to inform practice, different types of evidence are important for distinctive purposes 'based on three types of evidence that practitioners might seek, use and act on when considering 'what works' in health and social care: theoretical evidence, empirical evidence and experiential evidence' (2007b, 434). They defined these as follows: theoretical, 'Ideas, concepts and models used to describe the intervention, to explain how and why it works and to connect it to a wider knowledge base and framework'; empirical, 'Information about the actual use of the intervention and about its effectiveness'; and experiential, 'Information about people's experiences of the service or intervention and the interaction between them'. Glasby, Walshe and Harvey then explain how each of these makes a specific contribution to knowledge (2007b, 434).

Building on the experiential aspect of evidence, Francis-Auton et al. in their state-of-the-art review of understanding the 'experience' in EBCD, stress the importance of experiential knowledge for designing user-centred healthcare and that the real challenge lies in making healthcare experience methods accessible (2024, 11). Design has much to offer by way of eliciting responses from individuals' experiences as useful evidence. Prototypes are one method used in design both to probe for insights and ideas and as a springboard for more speculative and futures-oriented propositions. This discussion is well-rehearsed in Coughlan, Fulton-Suri and Canales who describe prototyping as a means of 'building to think', for making the intangible 'tangible, created so everyone can grasp the idea', and where prototypes are offered as ''transitional objects' … objects that support a change from a current behavior to a new behavior.' Prototypes, they argue, are able to elicit forms of evidence through the testing of hypotheses, 'bringing … insights to the surface' (2007, 127–131). Sanders and Stappers continue this line of argument discussing designers' ability to 'make things that describe future objects', and for using prototyping which 'allows people to experience a situation that did not exist before.' (2014, 6).

Design strategist Hagen states 'much is to be gained from effective integration of evidence-based and user experience-based approaches to design for healthcare services' (Hagen 2014, quoted in Robert and Macdonald 2017, 127). When discussing evidence-based medicine, Carr et al. also call for integrating experience-based design along with evidence-based design,

Integrating an [experience-based design] approach involves far more than asking patients how they 'felt' about a service or building. It moves consultation to a new level of co-design, and even co-production, in the process building a sense of community and ownership around a project. (2011, 30).

As for theoretical evidence, Sales et al. highlight the issue of the partial—or lack of -success in implementing evidence-based practices due to the loose application of theory and provide an example of linking theory to intervention design for a mental health application, discussing the 'careful consideration of theory in planning to implement practice-based practices into clinical care' (2006). Additionally, the theoretical basis underlying the different approaches to designing and implementing interventions might be relatively or completely unknown outside of a particular field, e.g. the use of visualisation theory (e.g. Padilla et al. 2018; Tsattalios 2017) commonly used in design, may be unfamiliar to some fields across health.

## Towards a cognitive framework for design and health collaborations

**Figure 3.**
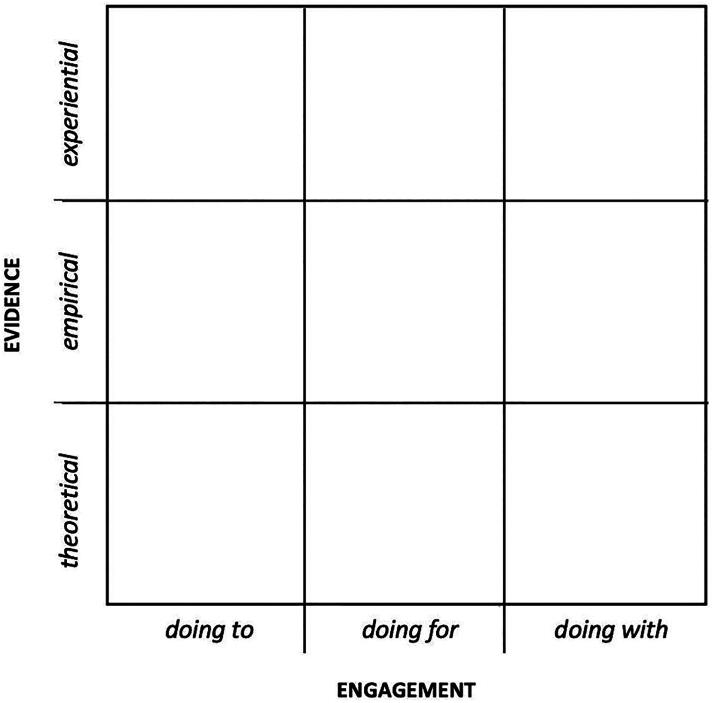
The proposed matrix for mapping the design/health research space, based on the two axes of evidence (experiential, empirical and theoretical) and engagement (doing to, doing for and doing with).

With reference to the modes of engagement discussion above, the distinction between and separation of 'expert mindset' from 'participatory mindset' in Sanders' model now appears problematic, particularly when 'a key tenet of co-design is that users, as 'experts' of their own experience, become central to the design process' (Design for Europe n.d.). This was a point perhaps conceded earlier by Sanders in her frequently cited reference to those who buy and use products and services as 'virtuosos of the experience domain' (Sanders 2001). However, common to both design and health research approaches has been the acknowledgement of different levels and modes of engagement, depending on intent. Borrowing from the Arnstein (1969) and NEF (2014) models described above, the author proposes that 'mode of engagement' would form a useful, horizontal X axis. Using this revised arrangement, the horizontal axis still aligns conveniently with Sanders' model, with its subject focus ('doing to') to the extreme left of the axis, and participant focus ('doing with') to the extreme right. Implicitly overlaying this axis is the differentiation of hierarchical (left) from more democratised (right) approaches. As for evidence, as discussed above, the separation of 'design-led' from 'research-led' is now potentially problematic as some design techniques are recognised as legitimate methods for eliciting valuable evidence. For instance, those employed in Human-Centred Design (HCD) and used in health services design (HSD), such as participative, iterative and creative approaches and processes, ensure users' views and needs—informed through their experiences—are kept to the fore throughout the services innovation process eliciting valuable evidence of their needs (Fischer et al. 2021). If empirical, experiential and theoretical evidence are accepted as having equal weight, and design-led (research) methods can also elicit these, then the distinction between research-led and design-led becomes blurred. In setting out the new framework, the author proposes that the vertical Y axis is, instead, one concerned with evidence, employing the equally weighted categories proposed by Glasby, Walshe, and Harvey (2007b), i.e. theoretical, empirical and experiential. Together, these two axes now provide the basis for a topographic framework modelled as in Figure 3.

## Discussion

### A global healthcare challenge

The escalating threat posed by antimicrobial resistance (AMR) (O'Neill 2016), where infection prevention and control (IPC) has a vital role to play, is one example of a global health challenge prompting increasing calls for TDR. However, despite best endeavours by the IPC community, effective IPC compliance appears to remain problematic and of particular concern (e.g. Schutte et al. 2024) despite often mandatory training. IPC training content and approaches have tended to be determined predominately by specialists in the IPC and microbial communities. However, as IPC is 'everyone's business' (Sutton, Brewster, and Tarrant 2019) and for IPC measures to be effective, they need to be adopted by a variety of players on a broad front across diverse settings, not just by those receiving specialist IPC training. So, might some TDR approaches, employing less familiar combinations of expertise, forms of engagement and evidence, including those from design, prove useful for a broader range of purposes and audiences? Could a mapping framework, such as the example proposed above, assist here?

### Diverse approaches to training for infection prevention and control

Rather than discuss hypothetical examples, four approaches to developing IPC training resources, drawing from existing examples or published, peer reviewed pilot studies, are now briefly discussed with reference to the framework and considered from a TDR perspective. (Full details of each example are limited here by space but are available *via* the associated references.) The intention is, through these examples, to illustrate potentially useful differences in approach to—and combinations of—theory, types of evidence and engagement used, and the different expertise required for each, to help address the challenge on a broader front.

## Example 1: 'Conventional' approaches for specialist staff

In 'conventional' approaches to IPC training intended for hospital staff as part of their professional development, the approach and content is typically determined by expertise from a relatively narrow range of specialisms, i.e. the microbial sciences and IPC. Training content would tend to draw from empirical lab-based data, and empirical and experiential evidence gained from the containment and management of infection outbreaks. This content is usually structured into online courses *via* flexible modules for staff to work through, perhaps from basic to advanced levels, to attain recognised certification, sometimes requiring a substantial investment in time. To enhance engagement, content is often contextualised through case studies or scenarios that promote an understanding of correct protocols, such as appropriate hand hygiene or disinfection regimes and may incorporate videos, interactive and gamification elements, workbooks, and online quizzes. Although their intention is often to achieve some form of behaviour change, Schutte et al. (2024) identify a lack of underpinning theory for implementing these types of intervention. Engagement would perhaps involve little or no co-development with intended users, so limited to 'doing for'. ARHAI (2023), NES (2024); IPS (2024), and GAMA Healthcare (2024) typify this type of approach.

### Example 2: Experiential practice-based learning for specialist staff

There is a significant difference between learning and being tested online for appropriate IPC protocols and applying these effectively in practice. An innovative approach to IPC training for specialist staff is to adopt experiential, practice-based learning within a simulated practice environment. One example is King et al. (2024) study which utilised Kolb's 4-stage learning cycle theory (Kolb 1984, quoted in Wijnen-Meijer et al. 2022). This approach, following pre-requisite online IPC training, intended to engage specialist infectious disease trainees and IPC professionals in a simulated outbreak mirroring a real-life incident. Although the range of expertise would appear largely familiar in the IPC training sector (the study involved IPC and microbial sciences specialists), this example makes use of Kolb's 4-stage theoretical underpinning (concrete experience, reflective observation, abstract conceptualization, and active experimentation), taking a more participative, experiential and reflective 'learning by doing', and 'doing with' approach whose efficacy was evaluated through combining both quantitative and qualitative data collection methods. Feedback highlighted the realism and educational value of simulation. Results evidenced significant improvements in participants' knowledge and confidence in outbreak management, and a positive impact on their practice.

### Example 3: Visualisation and co-design with non-(IPC) specialist hospital staff

Whereas examples 1 and 2, whose approach and content was predominantly determined by IPC and microbial sciences expertise, and which were largely concerned with training specialist IPC and key professional staff, example 3 adopted a more pluralistic, participative, action-research approach to explore what might form effective IPC training content for a broader, less specialist range of intended trainees, in this case, non-IPC-specialist ward-based staff, including junior doctors, nurses and domestic staff. With the contemporary policy turn towards co-production, in addition to microbial and nursing expertise, the team included expertise to implement the co-development and evaluation of a tablet-based training app employing a virtual ward model (requiring software and programming expertise) through an iterative, design-led prototyping process (requiring co-design expertise). Key principles of IPC were conveyed through a novel 'making visible the invisible' approach showing the characteristics, routes of transmission and persistence of named pathogens in the face of various ward IPC cleaning regimens. This drew from evidence of the value of visual approaches in healthcare interventions (e.g. Galmarini, Marciano, and Schulz 2024). Results from this pilot study reported changes in perception of infection risk, the benefits of a more contextualised understanding of the content and of the potential for visual apps to be used in IPC training particularly in understanding how to engage (non-IPC-specialist) staff who have little time to invest in IPC training (Macdonald et al. 2017).

### Example 4: Narrative techniques for non-(IPC) specialist veterinary staff

Example 4 built on example 3's visual-, and codesign-led expertise, this time developing an app for training non-IPC-specialist veterinary staff with little time to invest in deep IPC training. As in examples 1–3, the team included and benefitted from significant input from microbial sciences and IPC expertise (here, from the veterinary field), but these played more of an implicit role with no specific pathogens being named, focussing more on 'making visible' potential reservoirs of infection, routes of transmission and risky behaviours during a surgical procedure. Adopting a narrative approach (e.g. Greenhalgh, Russell and Swingelhurst 2005) to develop a 'serious health story' (Lugmayr et al. 2017), a set of scenarios of a companion animal undergoing a specific surgical procedure were co-developed with a range of veterinary staff. These were conveyed through an interactive digital model of a veterinary referral practice and embodied elements of serious games theory (Maheu-Cadotte et al. 2021). The intention was to identify risk, to change perception of risk as a prompt to change behaviour, often the focus of improvement interventions (Greene and Wilson 2022). Results from the pilot study reported changed perception of risk and intent to adopt improved IPC measures (Macdonald et al. 2023).

### Transdisciplinary research (TDR)

TDR is an approach increasingly recognised as vital in tackling particularly problematic challenges. In UKRI-related TDR calls, at least three fields of expertise associated with different UK research funding councils are required to be represented in proposals. Yeung et al. define three characteristics of a TDR undertaking as one which,

1) seeks to integrate knowledge and perspectives from diverse backgrounds to come to a shared and more sophisticated understanding of the problem at hand; 2) attends to relationship building and communication in ways that transform, re-conceptualize and extend ideas, methods and theories; and 3) encourages co-creation to rework and implement novel and feasible solutions. (2021, 1-2)

**Figure 4.**
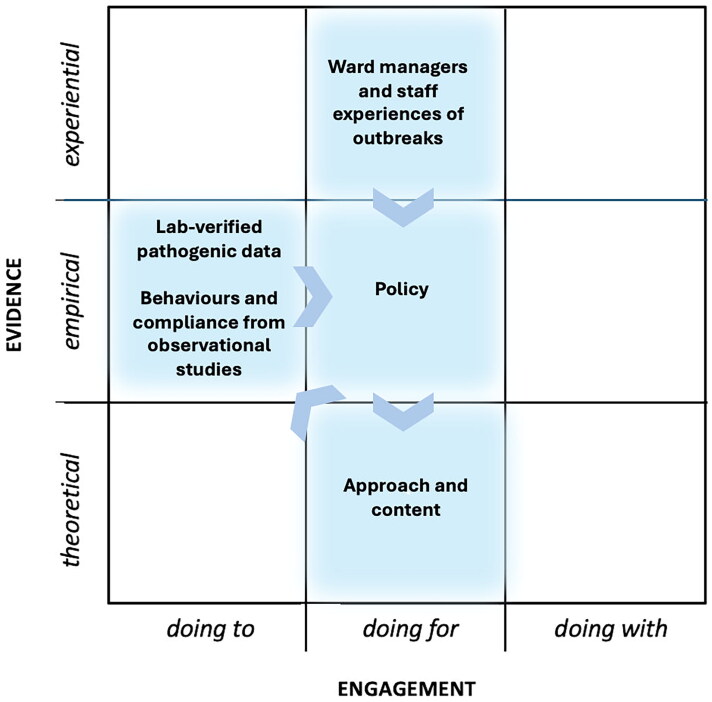
'Conventional' content and approach to IPC training materials, devised by specialist leads in IPC and microbial sciences, normally intended for specialist staff and as part of their professional development. Empirical evidence from lab data, observational studies and outbreak management informs policy and training content. Little co-creation. Engagement limited to participation in training.

Having proposed the mapping framework above, could reference to this and Yeung et al. (2021) three characteristics of TDR potentially help prompt consideration of unconventional, less familiar groupings of expertise, each with their own proclivity for types of evidence and engagement, to address particularly challenging issues on a broader front? Example 1 (Figure 4) maps clearly onto the 'empirical', and 'doing to—doing for' spaces, drawing also from some 'experiential' knowledge. However, in example 1, none of the three TDR characteristics would appear to be met.

**Figure 5.**
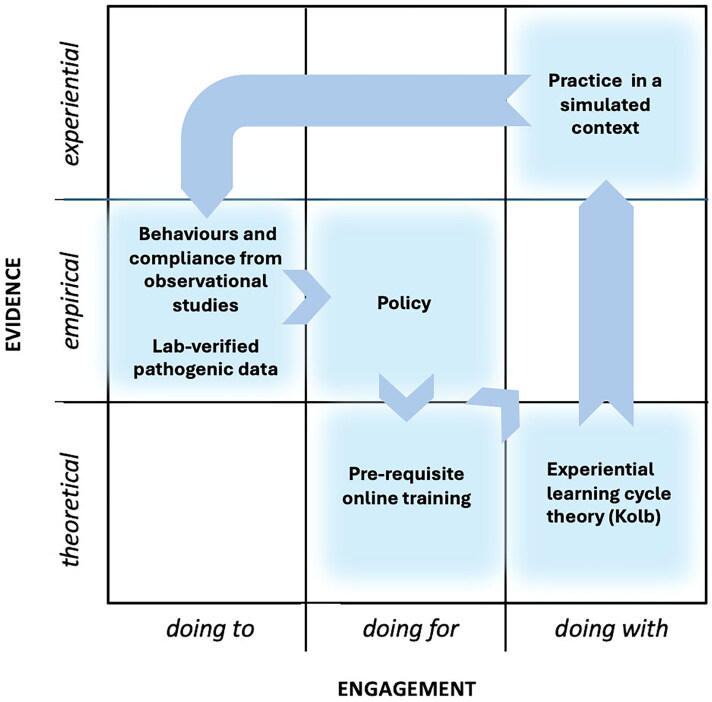
Kolb's experiential learning cycle provides the theoretical basis for experiential practice-based learning. Participants have the opportunity to 'rehearse', discuss and evaluate the outcomes of appropriate practice, actions and protocols in a safe, simulated environment.

Example 2 (Figure 5) extends example 1's more conventional IPC training approaches by adopting a more 'experiential' and 'doing with' approach through participation in a simulated outbreak derived from 'empirical' evidence. Also in example 2, while the expertise involved appears similar to that in example 1, it takes a novel approach by adopting learning theory (moving closer towards Yeung et al. [2021] characteristic 1), and there is a stronger emphasis on the type of communication and extending of methods and theories employed (characteristic 2). However, there appears little evidence of co-creation (characteristic 3).

**Figure 6.**
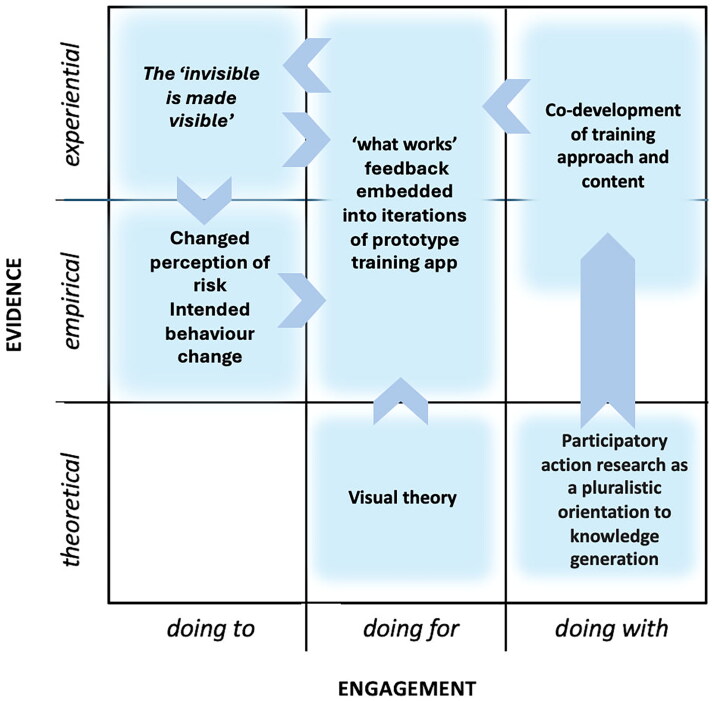
Visual theory and visualisation are used to 'make visible the invisible'. Together with a participative co-design and iterative prototyping approach, incorporating 'what works' at each stage to enhance content appropriate for engaging non-specialist healthcare staff.

**Figure 7.**
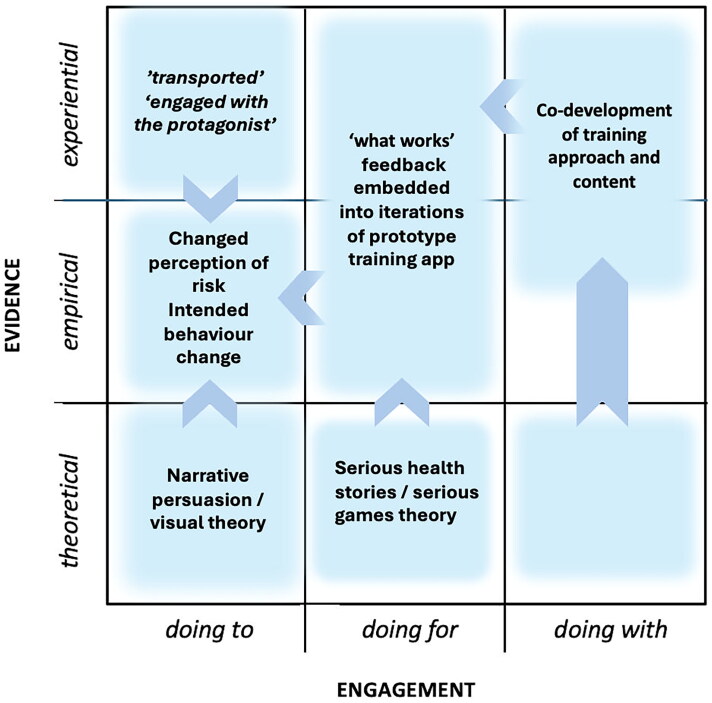
Visual, narrative persuasion and serious health gaming theories are brought together in this study. Similarly to example 3, an iterative prototyping and co-design process incorporates 'what works' at each stage to enhance content appropriate for engaging non-specialist healthcare staff.

Examples 3 and 4 (Figures 6 and 7) broaden the range of collaborating expertise normally associated with developing IPC training to include a 'non-typical' (in IPC training) field, design. Design brings with it its use of visual theory (which, it is argued, provides a useful basis for behaviour change interventions (Hinyard and Kreuter 2007; Williams et al. 2012; Murray et al. 2016)). Examples 3 and 4 also take a more participative 'doing with' approach using 'experiential' evidence of what might be effective for the engagement of their particular audiences, the feedback from these 'virtuosos of experience' (Sanders 2001) being embodied into each subsequent iteration of the training prototype throughout the co-development process. Example 4 extends the theoretical base further through employing narrative theory, and serious storytelling, and also using gaming theory approaches familiar in user experience (UX) design fields. Examples 3 and 4 both fulfil the three characteristics of TDR (table 1).

**Table 1.** How each of the four examples 1 to 4 map on to Yeung et al. (2021) three characteristics of transdisciplinary design.

Example Yeung et al. (2021) Three Characteristics of Transdisciplinary Research (TDR)	1	2	3	4
1) seeks to integrate knowledge and perspectives from diverse backgrounds to come to a shared and more sophisticated understanding of the problem at hand	**✗**	**?**	✔	✔
2) attends to relationship building and communication in ways that transform, re-conceptualize and extend ideas, methods and theories	**✗**	✔	✔	✔
3) encourages co-creation to rework and implement novel and feasible solutions	**✗**	**✗**	✔	✔

## Conclusion

Sanders' seminal map has proved a useful point of reference for many in design, a visual and shareable cognitive map for provoking conversations about the relative positioning of different research methods within the design research landscape. Her map suggests further possible forms and uses of cognitive maps in design such as the one proposed here.

If IPC is 'everyone's business' (Sutton, Brewster, and Tarrant 2019), each of the approaches in the four approaches to IPC training examples above, intended for different audiences and purposes, has a potential role and value for tackling IPC training on a broader front than is currently being taken. It is through calls such as the UKRI TDR calls that disciplines normally unfamiliar with one another will be required to work together in a TDR fashion. Currently, approaches such as those in example 1 are the only ones to have been extensively adopted and evaluated in the field for IPC compliance and for the extent to which they reduce the causes and transmission of infections. The more novel approaches such as in 2, 3 and 4 are yet to be adopted, scaled and thoroughly evaluated for their potential efficacy. However, if the three characteristics of TDR described above are being increasingly sought in UK funding calls to tackle complex, sometimes intractable and here, health-related, issues on a broader front, there appears to be a need, particularly at the conceptualisation and planning stages, for tools which help suggest new, perhaps less familiar alliances and synergies between disciplines.

The suggested mappings in the four examples above hopefully illustrate that other, key audiences need to be considered over and above those conventionally considered for IPC training and that these may benefit from design's input as part of a TDR study. The mapping framework proposed here is offered as one version of such a tool, a 'visuospatial layout' where design's potential contributions could be made more explicit and 'externalized' (Shen, Tan, and Siau 2019, 281), to provide the prompts for discussion promoting diverse, yet complementary modes of engagement and types of evidence. While readers may take issue with the author's particular approach to the mappings using the framework, it is offered in the same spirit as Sanders', as a 'scaffolding for thinking and talking about design research'; she invited the design research community to engage in a collective conversation prompted by her map. It is hoped that, through the suggested mappings and the above examples, design's potential contribution in terms of useful theory, types of engagement and evidence in the TDR design/health space highlighted here can be explored and critiqued in further research.
